# Peripheral direct current reduces naturally evoked nociceptive activity at the spinal cord in rodent models of pain

**DOI:** 10.1088/1741-2552/ad3b6c

**Published:** 2024-04-17

**Authors:** Tom F Su, Jack D Hamilton, Yiru Guo, Jason R Potas, Mohit N Shivdasani, Gila Moalem-Taylor, Gene Y Fridman, Felix P Aplin

**Affiliations:** 1 School of Biomedical Sciences, University of New South Wales, Sydney, New South Wales, Australia; 2 Eccles Institute, John Curtin School of Medical Research, The Australian National University, Canberra, Australian Capital Territory, Australia; 3 Graduate School of Biomedical Engineering, University of New South Wales, Sydney, New South Wales, Australia; 4 Department of Otolaryngology, Head and Neck Surgery, Johns Hopkins University, Baltimore, MD, United States of America; 5 Department of Biomedical Engineering, Johns Hopkins University, Baltimore, MD, United States of America; 6 Department of Electrical and Computer Engineering, Johns Hopkins University, Baltimore, MD, United States of America

**Keywords:** peripheral neuromodulation, direct current neuromodulation, implantable neuromodulation, chronic pain, neuropathic pain, inflammatory pain, *in vivo* electrophysiology

## Abstract

**Objective.:**

Electrical neuromodulation is an established non-pharmacological treatment for chronic pain. However, existing devices using pulsatile stimulation typically inhibit pain pathways indirectly and are not suitable for all types of chronic pain. Direct current (DC) stimulation is a recently developed technology which affects small-diameter fibres more strongly than pulsatile stimulation. Since nociceptors are predominantly small-diameter A*δ* and C fibres, we investigated if this property could be applied to preferentially reduce nociceptive signalling.

**Approach.:**

We applied a DC waveform to the sciatic nerve in rats of both sexes and recorded multi-unit spinal activity evoked at the hindpaw using various natural stimuli corresponding to different sensory modalities rather than broad-spectrum electrical stimulus. To determine if DC neuromodulation is effective across different types of chronic pain, tests were performed in models of neuropathic and inflammatory pain.

**Main results.:**

We found that in both pain models tested, DC application reduced responses evoked by noxious stimuli, as well as tactile-evoked responses which we suggest may be involved in allodynia. Different spinal activity of different modalities were reduced in naïve animals compared to the pain models, indicating that physiological changes such as those mediated by disease states could play a larger role than previously thought in determining neuromodulation outcomes.

**Significance.:**

Our findings support the continued development of DC neuromodulation as a method for reduction of nociceptive signalling, and suggests that it may be effective at treating a broader range of aberrant pain conditions than existing devices.

## 1. Introduction

Chronic pain, a debilitating condition affecting roughly one in five people [1], is defined as pain that persists for at least 3 months [2]. Chronic nociceptive pain is caused by damage or inflammation in non-nervous tissue, while chronic neuropathic pain is caused by lesions within the somatosensory system itself [3]. First-order drugs for both subtypes of chronic pain are not well suited to chronic prescription due to drawbacks such as high dependence risk in opioids [4, 5] or gradual loss of efficacy in gabapentinoids [6]. Electrical neuromodulation devices such as conventional spinal cord stimulators, high frequency spinal cord stimulators, or peripheral nerve stimulators offer a non-pharmacological alternative for chronic pain management [7–10]. Although many such devices have received clinical approval, they only provide relief for a subset of patients [11, 12], and there is limited evidence to support their efficacy for chronic nociceptive pain specifically [13, 14]. A potential explanation for these shortcomings lies in the use of short electrical pulses for neuromodulation, which primarily affect large-diameter (typically non-nociceptive) A*α*/A*β* fibres. It is generally understood that conventional spinal cord stimulators leverage spinal gating mechanisms to indirectly modulate nociceptive signalling [15, 16], although they may also affect spinal cord signalling in other ways [17, 18]. The mechanisms of action of newer kilohertz-frequency spinal cord stimulators are less well understood and may not involve gating mechanism. There is, nevertheless, evidence that these devices are also reliant on interactions with large-diameter fibres [19–21]. An alternative neuromodulation paradigm which instead targets the activity of small-diameter, nociceptive A*δ*/C fibres could therefore allow devices to provide pain relief in a wider range of contexts without interfering with innocuous sensations necessary for everyday life.

Direct current (DC) neuromodulation may provide this alternative, as there is a growing body of evidence suggesting that small-diameter neurons are sensitive to DC and low frequency waveforms [15, 16]. DC has historically been avoided in clinical devices due to cytotoxicity concerns [22, 23], but recent engineering developments have paved the way for safe *in vivo* DC delivery [24–26]. Indeed, neuropathic pain relief using ultra-low frequency dorsal column stimulation has been demonstrated in a recent clinical trial [27]. Another approach using peripheral DC wherein nociceptive signalling is selectively disrupted before reaching the spinal cord has also been proposed [16, 26, 28]. This could be particularly beneficial for the treatment of chronic nociceptive pain (e.g. post-surgical or arthritic pain), which is not well managed by existing neuromodulation devices [13, 14].

However, it is unknown if peripheral inhibition of small-diameter fibre activity translates to targeted reduction of activity in pain pathways. Previous studies characterised fibres by electrically evoked response latency rather than sensory input [16, 27]. While there is a correlation between response latency and function, this relationship is disrupted in chronic pain conditions where large-diameter A fibres contribute to pain hypersensitivity via mechanical allodynia and hyperalgesia [29, 30]. Clarifying the functional, rather than anatomical, populations targeted by DC is important for evaluating the therapeutic potential of peripheral DC neuromodulation. Given that chronic nociceptive and chronic neuropathic pain promote different pathological changes within the nervous system [31], it is also necessary to compare DC neuromodulation outcomes across both pain conditions.

Here, we compare the effects of peripheral DC neuromodulation in rodent models of neuropathic and inflammatory pain by recording spinal responses to functionally relevant peripheral stimuli. Based on previous findings which measured electrically evoked activity [16], we hypothesised that responses evoked by noxious stimuli would be preferentially reduced in all treatment groups. We found that noxious-evoked responses were reduced in all animals. Furthermore, we demonstrated that conduction block persisted for longer in nociceptive responses. Our findings support the continued translation of peripheral DC neuromodulation devices as a potential treatment for both nociceptive and neuropathic pain.

## 2. Materials and methods

### 2.1. Animals

Experiments were performed on Sprague Dawley rats aged 10–23 weeks of both sexes (25 females, 235–387 g; 22 males, 453–720 g) obtained from the Animal Resources Centre (WA, AUS). Rats were housed on a 12 hour light-dark cycle with ad libitum access to food and water, in groups of up to four according to sex. All procedures were approved by the University of New South Wales Animal Care and Ethics Committee (20/80 A).

### 2.2. Pain models

#### 2.2.1. Neuropathic pain model

A neuropathic pain state was induced using the spared nerve injury (SNI) model described by Decosterd and Woolf [32], with the tibial branch of the sciatic nerve spared instead of the sural branch [33, 34]. Sparing of the tibial branch maintained hindpaw sensation across a larger dermatome whilst preserving neuropathic pain behaviours resulting from the injury. Rats were anaesthetised with isoflurane (4% induction, 1.5% maintenance) in oxygen (2 l min^−1^ induction, 1 l min^−1^ maintenance). The common peroneal and sural nerves on the left side were ligated with Mersilk 5/0 suture (Ethicon; NJ, USA) and axotomised distally, while the tibial nerve was left intact. The wound was sealed and antiseptic cream applied. Rats were monitored until awake and behaving normally. General health and wellbeing were monitored daily following the procedure until the terminal electrophysiology experiments 8 d later, when peak pain hypersensitivity was achieved [32, 33].

#### 2.2.2. Inflammatory pain model

An inflammatory pain state was induced by subcutaneous injection of complete Freund’s adjuvant (CFA) [35]. Rats were anaesthetised as above and 100 *μ*l CFA (Sigma-Aldrich; MA, USA) was injected subcutaneously into the plantar surface of the left hindpaw to produce a local inflammatory reaction. Post-operative pain monitoring was performed as per neuropathic pain models, with the exception that the terminal experiment was instead conducted 5 d after CFA injection to correspond to peak pain hypersensitivity in this model [36].

### 2.3. Behavioural tests

Sensitivity to mechanical allodynia and thermal hyperalgesia were tested before injury to establish baseline, and again on day 7 for SNI-treated rats, or day 4 for CFA-treated rats; different time courses were chosen to allow peak pain hypersensitivity to develop in each injury [32, 33, 36]. Testing was performed after 15 min of habituation and in well-controlled environments to minimise non-specific responses [37]. Withdrawal reflexes were defined as sudden lifting and/or licking of the hindpaw in both tests.

To verify the development of mechanical allodynia, rats were habituated in an enclosure on an elevated mesh grid. Withdrawal threshold was measured by pressing an electronic von Frey aesthesiometer (IITC Life Science Inc.; CA, USA) into the plantar hindpaw until a withdrawal reflex was evoked [37]; the maximal force applied was recorded. Three such measurements were at 2 min intervals.

To verify the development of thermal hyperalgesia, rats were instead habituated in a glass-bottomed cage. A 50 mW cm^−2^ infrared heat source (heat-flux radiometer; Ugo Basile; VA, ITA) was applied to the plantar surface of the hindpaw until a withdrawal reflex was evoked and the withdrawal latency recorded [38]. A pre-determined cut-off latency of 30 s was used to prevent tissue damage. Three trials were performed at 2 min intervals.

### 2.4. Electrophysiology

**Figure 1.**
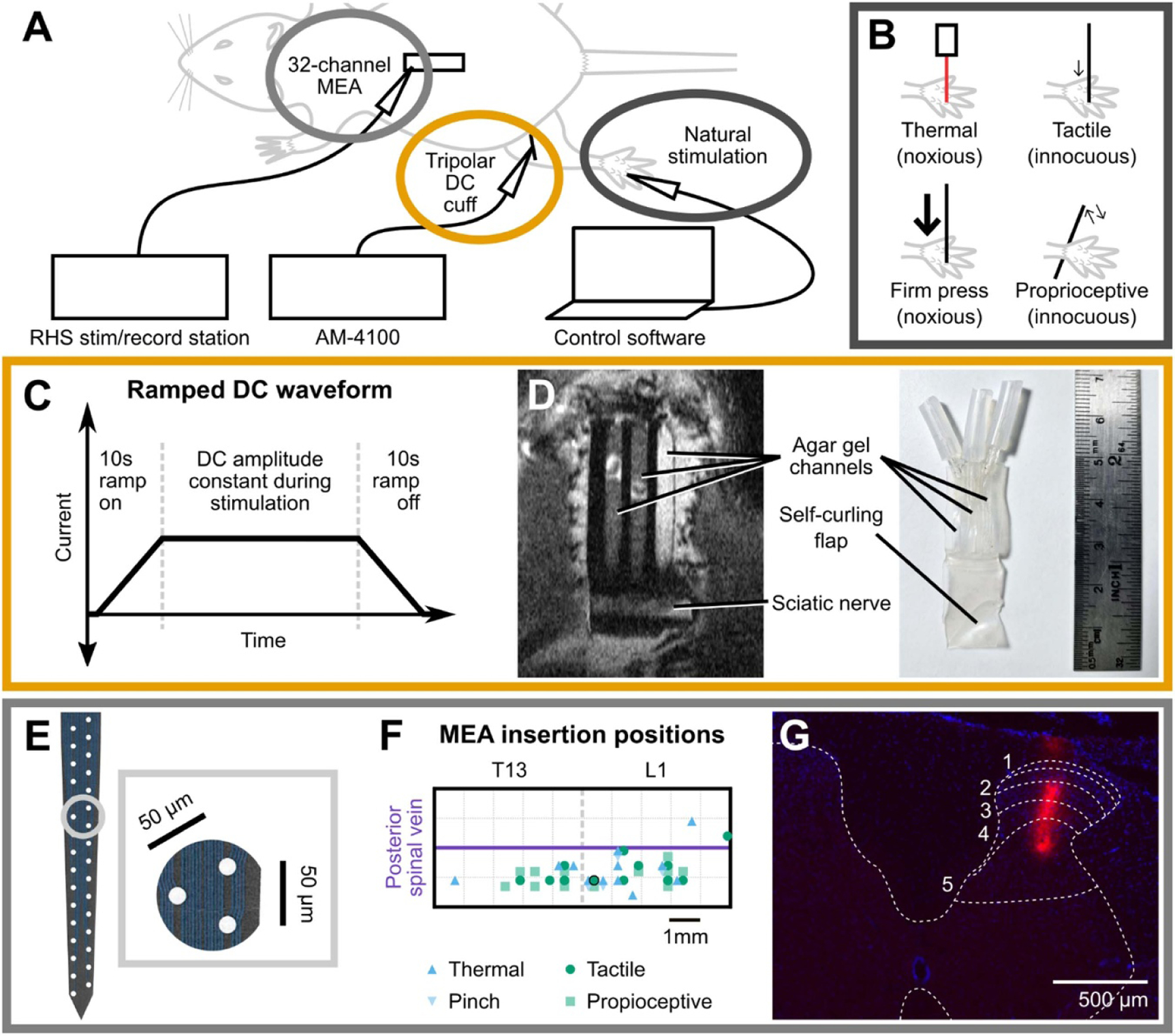
Experimental setup. (A) Electrophysiological setup. Spinal recordings were taken using a 32-channel MEA and an Intan RHS Stim/Record System. DC was delivered to the sciatic nerve by an AM-Systems stimulator via a tripolar cuff. The hindpaw was stimulated naturally with the aid of software triggers. (B) Stimulus types. Four natural stimuli were chosen to evoke responses that were noxious thermal-, noxious press-, tactile-, or proprioceptive-dominant. (C) Ramped direct current waveform delivered via tripolar cuff. (D) Tripolar silicone cuff used for DC delivery. A transverse MRI scan of an implanted cuff (left) is shown alongside a photo of a cuff pre-implantation (right). Note that there are three agar channels by which DC is delivered to the nerve, comprising a centre channel of opposing polarity to the two flanking returns. (E) MEA layout. NeuroNexus A1x32-Poly2–5 mm-50 s-177 MEA with 50 *μ*m electrode spacing. Inset: example three-channel set chosen for spike-sorting. (F) MEA insertion positions. MEA was inserted into T13/L1 spinal segments ipsilateral to the stimulation site. (G) DiI stain of MEA insertion highlighted in black. Red fluorescence shows depth and medio-lateral position. Dorsal horn and approximate L1 spinal laminae positions are marked. DC = direct current; MRI = magnetic resonance imaging; MEA = multi-electrode array.

Rats were anaesthetised using urethane (1.5 g kg^−1^ body weight; Sigma–Aldrich; MA, USA) administered via intraperitoneal injection. 10 ml kg^−1^ body weight of modified Krebs–Henseleit solution (117.9 mM NaCl, 4.7 mM KCl, 25 mM NaHCO_3_, 1.3 mM NaH_2_PO_4_, 1.2 mM MgSO_4_, 2.5 mM CaCl_2_) was injected subcutaneously every 3 h to maintain hydration. The animal was placed on a heating block (36.5 °C) and intubated by tracheotomy. As shown in figure 1(A), the animal was fixed to a stereotaxic frame (Stoelting; IL, USA) for collection of electrophysiological data, with natural stimulation delivered to the foot (figure 1(B)), DC delivered to the sciatic nerve (figures 1(C) and (D)), and multi-unit recordings made at the spinal cord (figures 1(E)–(G)). A laminectomy was performed to expose the T13 and L1 regions of the dorsal spinal cord. The dura mater and arachnoid mater covering the exposed spinal cord were removed, and a 32-channel penetrating multielectrode array (MEA; NeuroNexus; MI, USA) was inserted using a micromanipulator ipsilateral to the stimulation site (figures 1(E) and (F)). Voltage signals were band-pass filtered from 0.1 Hz to 20 kHz by an RHS Stim/Recording Headstage and recorded at 30 kHz using an RHS Stim-Recording system (Intan Technologies; CA, USA). To visualise the depth of electrode penetration, the MEA was soaked in DiI (Sigma-Aldrich; MA, USA) before insertion into the spinal cord in three animals (figure 1(G)). At the end of the experiment, those animals were perfused via 4% paraformaldehyde and their spinal cords removed, cryoprotected, and sectioned at 10 *μ*m on a Leica CM1950 cryostat (HE, DEU). Sections were imaged using an Olympus IX83 Inverted Microscope (MA, USA).

### 2.5. Peripheral stimulation

Natural stimuli of four modalities (noxious thermal–, noxious pinch-, tactile-, and proprioceptive-dominant) were delivered to the left hindpaw of the animal as described below. When multi-unit spinal responses were obtained, the modality of its components was first qualitatively determined by briefly applying tactile-, proprioceptive-, noxious pinch-, and noxious thermal–dominant stimulus in that order. Note that some units were found to be multimodal. Following determination of modality, stimuli were presented in order of relevance and the responses recorded.

#### 2.5.1. Noxious thermal–dominant stimulus

To evoke a thermoreceptive-dominant response, a 980 nm continuous wave diode laser (Changchun New Industries Optoelectronics Technology Co., Ltd; JL, CHN) with a beam diameter of 4 mm was positioned normal to the plantar surface of the hindpaw. A 1 s beam was given at 1.95–6.85 W to heat the stimulation site by ~10 °C, as verified by a ThermaCAM Reporter7 Pro thermal camera (FLIR Systems; OR, USA). Sets of two laser pulses were recorded, with an inter-pulse interval (resting period between laser pulses) of either 20 s (*n* = 66 recordings) or 5 min (*n* = 209 recordings).

#### 2.5.2. Noxious press–dominant stimulus

To evoke a high-threshold (noxious) mechanoreceptive-dominant response, a custom piezoelectric pressure sensor was fixed to the table and the hindpaw placed upon it with the plantar surface facing upwards. A wooden rod was used to firmly press the stimulation site at a rate of ~1 Hz. Sets of 50 stimulations were recorded alongside analogue voltage changes at the piezoelectric sensor [39].

#### 2.5.3. Tactile-dominant stimulus

To evoke a low threshold (innocuous) mechanoreceptive-dominant response a lightweight aluminium rod with a rounded plastic cap was fixed to a SignalForce V4 shaker (Data Physics; CA, USA) and positioned with its tip resting on the plantar surface of the hindpaw. Mechanical stimulation waveforms were passed through a SignalForce PA100E linear amplifier (Data Physics; CA, USA) to the shaker, which was calibrated for a peak downwards displacement of 100 *μ*m over 250 *μ*s using a laser displacement sensor (Micro-Epsilon Messtechnik; BY, DEU). Mechanical stimulation was given at a rate of 1 Hz and recorded as sets of 50 stimulations.

#### 2.5.4. Proprioceptive-dominant stimulus

To evoke a proprioceptive-dominant response, a wooden rod attached to a stepper motor (Pololu Corporation; NV, USA) was placed under the hindpaw, supporting the ankle. The motor rotated 15° over a 200 ms period before returning to its initial position, causing bilateral rotation of the ankle and knee joints. Sets of 50 rotations, delivered at 1 Hz, were recorded.

### 2.6. DC neuromodulation

The ipsilateral biceps femoris and gluteus superficialis were separated to expose a section of the sciatic nerve ipsilateral to the stimulation site [40]. A silicone cuff consisting of three electrolytic channels (a central active channel and two flanking returns) was inserted around the sciatic nerve. The channels were filled with 4% agar gel made using modified Krebs–Henseleit solution (see Electrophysiology above). A tripolar cuff design was selected to minimise onset activation [41]. While the cuff is active, the centre channel delivers current of one polarity and each return channel delivers current of the opposite polarity at half the amplitude. Cuffs were measured to have an impedance of 50–100 kΩ, allowing current delivery of up to 1 mA using a 100 V stimulator. The cuff ensured close and consistent contact with the sciatic nerve, reducing potential variability in the DC delivery site.

The channels were connected to a Model 2200 or 4100 isolated stimulator (A-M Systems; WA, USA) using stainless steel leads suspended in syringes of saline. This saline acted to spatially separate the metal interface from the animal, impeding the diffusion of electrolytic products towards the interstitial fluid. Additionally, the syringes and stainless steel leads were easily replaceable mid-procedure, providing another avenue for minimising the exposure of the sciatic nerve to cytotoxic ion species. Other electrode designs employing a similar principle have been demonstrated to allow safe *in vivo* DC delivery for periods of at least 1–4 h at comparable amplitudes [42, 43]. This acute experimental setup enables investigation of the effects and potential applications of DC while a chronically implantable DC stimulator is still under development [24].

A DC waveform with 10 s on-/offset ramp and a total duration 100–110 s was delivered through the cuff (figure 1(C)). As previous studies have already rigorously examined the relationship between DC amplitude and spinal activity, we chose to deliver DC at fixed amplitude levels in all recordings. This procedure better reflects clinical application in that clinicians adjust neuromodulation intensity based on population responses and/or self-reported perceptual thresholds rather than single neuron recordings. It is therefore important to determine if an overall reduction of activity can be achieved under these conditions. Two amplitudes were used (500 *μ*A and 1000 *μ*A) for both cathodic (−) and anodic (+) centre channel polarities. Using previous findings [16] and our own pilot data, we chose DC amplitudes which we expected to be threshold and suprathreshold, respectively. Ramp durations were chosen based on pilot data to minimise onset excitation associated with high frequency changes in the electric field [44, 45]. There were three sequential experimental phases in relation to DC application: pre-DC recordings to establish baseline activity, during-DC recordings to determine level of reduction from baseline, and post-DC recovery recordings to observe return to baseline. For the during-DC phase, the four permutations of DC amplitude and centre channel polarity were delivered in a pseudorandom order. Recordings and peripheral stimulation began after allowing the DC waveform to complete its onset ramp and concluded before the commencement of the offset ramp. MRI images were collected from a separate cohort of two animals that were implanted with the tripolar silicone cuff and allowed to recover over the course of 1 week (figure 1(D)). Images were taken at the Biological Resources Imaging Laboratory (UNSW; NSW, AUS) under isoflurane anaesthesia.

### 2.7. Spike-sorting and cell classification

Signal processing of electrophysiological recordings was performed using custom Julia scripts (v1.8) [46, 47]. Signals were band-pass filtered from 300–5000 Hz and separated into trials. Groups of three spatially adjacent channels were extracted for further analysis based on the clear presence of evoked spike activity. A pre-DC recording from each recording set and channel group was designated as the template and the principal components of its concatenated waveforms clustered using an unsupervised *k*-means algorithm [48]. When 1% or more inter-spike intervals for a given cluster were ⩽1 ms, it was isolated and reiterated through the *k*-means algorithm; this was performed up to four times per cluster before exclusion. The final clustering solution was then applied to other recording sets from the same response. Units were classified according to the stimulus paradigm delivered in the template. Noxious press—and proprioceptive-dominant stimuli unavoidably contained a tactile component and so sometimes evoked low-threshold tactile responses. To avoid unit misclassification due to stimulus overlap, we excluded multimodal responses from our analysis. For example, non–tactile-dominant units were discarded if they also showed a significant response to tactile-dominant stimulus.

To determine response windows for spike event analysis, relative frequency histograms were generated by collating spike timings across all recordings and experiments (figure S1). A separate histogram was calculated for each stimulus type, as these paradigms varied in both stimulus length and the latency of expected spike arrival times at the dorsal horn [49]. Baseline frequency was defined as the average firing rate during a pre-stimulus window. The response window was subsequently determined by finding the first and last post-stimulus bin with a frequency of at least 1.5 times the baseline frequency. Windows were rounded away from the peak frequency by one significant digit. The final window timings for each stimulation paradigm are displayed in table 1. Previous studies have reported a sustained elevated response to thermal stimulation after cessation of the stimulus [50, 51]; we therefore also analysed a second window for noxious thermal responses from the end of the stimulus response window (1.5 s post–stimulus onset) to the end of our recording block (4.5 s post–stimulus onset). For this condition, as we did not have 3 s of pre-stimulus data, a 1 s pre-stimulus window was used and spike counts were multiplied by 3. Spike events per recording set in pre- and post-stimulation windows were then counted and exported for statistical analysis. Responses were classified as ‘evoked’ if they had significantly more mean spike events in their post-stimulus window than pre-stimulus window (student’s *t*-test; *α* = .05).

### 2.8. Spontaneous activity

To determine the effect of DC on spontaneous activity, two distinct analyses were performed. Firstly, in each recording block, spike events (before spike-sorting), prior to the presentation of any stimuli, were extracted from all MEA channels and considered as multi-unit activity. Average spike rate for each channel was then calculated for recordings before and during DC application. Secondly, pre-stimulus spike rates were extracted from individual spike-sorted units that had passed the evoked criteria (see Spike-sorting and cell classification above), before and during DC application. Both of these measures were analysed using linear mixed effects regressions (LMERs) as described below in *Statistical analysis*. These two measures enabled analysis of spontaneous activity both broadly across the recording array and in the specific units used in the main analysis, respectively.

### 2.9. Statistical analysis

Custom R scripts (v4.2.2) were used for statistical analysis. For behaviour, measurements were analysed using linear mixed-effects regression with Satterthwaite’s method (LMER; *lmer* function from the *lmerTest* package in *R*) and analysis of variance (ANOVA) to determine significant differences between estimated marginal means (EMMs) (*α* = .05). Fixed effects included experimental phase, treatment group, and sex. Animal identifiers were used as a random effect. We chose this method of statistical analysis as it accounts for the nested data structure, repeated measures design, and presence of random effects in our datasets. An ANOVA analysis on raw means in this case would be inappropriate as assumptions of observation independence would be violated, increasing the risk of type I errors.

For electrophysiological recordings, analysis was performed using the same LMER package, with a separate model for each stimulation paradigm. Fixed effects included experimental phase, treatment group, presence of evoked response, sex, DC amplitude, time since template recording, and pre-stimulus spike count. Animal and unit identifiers were used as random effects. Model fitting was assessed by residual plot inspection. Where significant effects were found, multiple comparisons analysis using Tukey *p*-value adjustments were made (*emmeans* function from the *emmeans* package in *R*). Data are visualised as EMMs with standard error (EMM ± SE). Effects of polarity were analysed using a separate LMER, with DC amplitude, treatment group, presence of response, sex, time since DC, and pre-stimulus spike count as fixed effects, and animal and unit identifiers as random effects. Latency of the response peak (the bin centre at which the maximum number of spikes occurred) was analysed in tactile-dominant units using Kruskal–Wallis and Hartigans’ dip tests. To compare the duration after DC cessation for which a lack of evoked activity persisted, we continued to record sets of peripheral stimulation following DC cessation. Recovery time was defined as the time relative to DC cessation of the first post-DC recording set which passed our evoked activity criteria. A Mann–Whitney U test and Wilcoxon rank-sum post-hoc analysis was performed on the recovery times of units which ceased to produce evoked activity during DC delivery (defined as no significant peri-stimulus compared to pre-stimulus activity as per our spike-sorting criteria), with a Bonferroni adjustment for multiple comparisons. All significance was defined as *α* = .05.

## 3. Results

**Figure 2.**
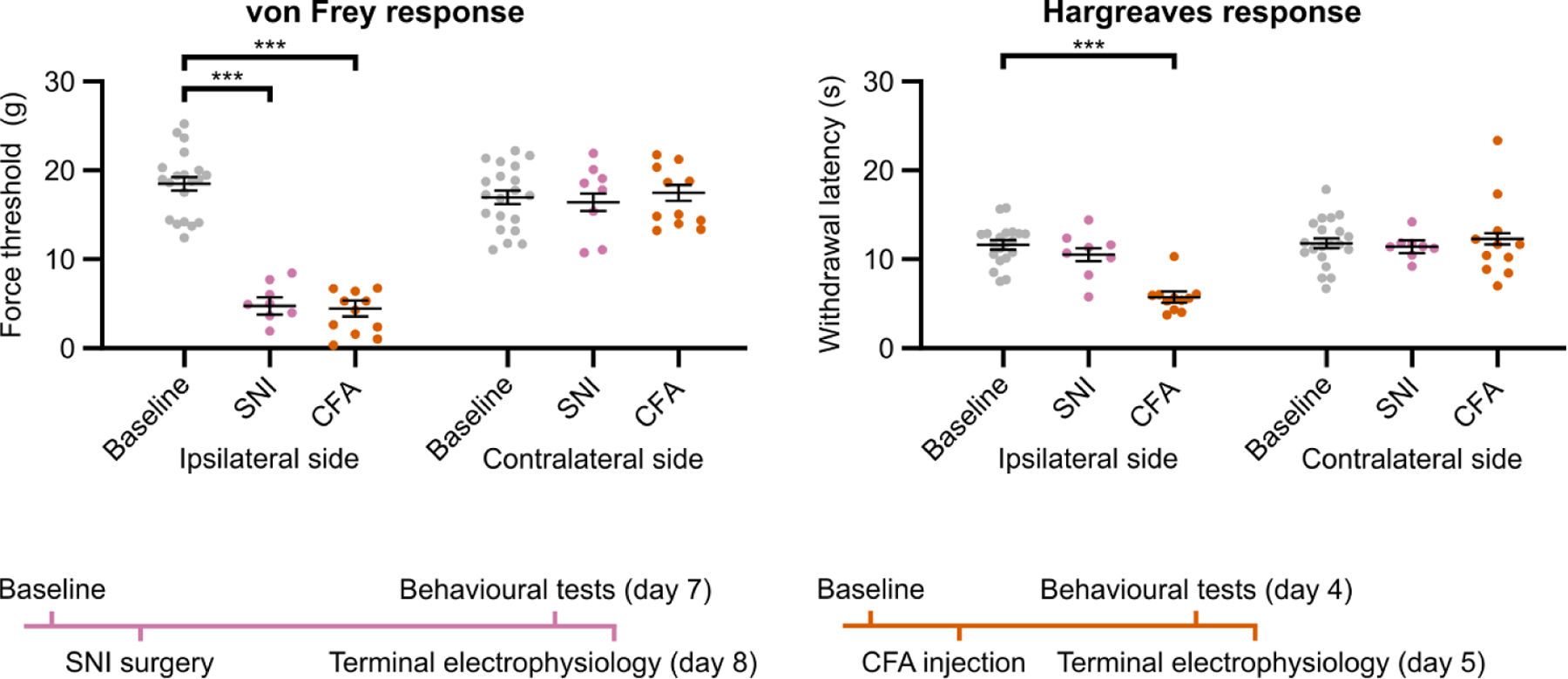
Behavioural verification of pain models. von Frey force thresholds and Hargreaves response latencies for both hindpaws in each treated animal, at baseline and one day before terminal experiment. Data were analysed using a LMER followed by ANOVAs to compare EMMs. Force thresholds for the ipsilateral hindpaw were significantly lower than baseline in all treated animals (*p* < .001), while response latencies were lower in the ipsilateral hindpaw of animals in the inflammatory pain model group only (*p* < .001). Time courses for each pain model are depicted below plots. EMM ± SE is shown. ***: *p* < .001. LMER = linear mixed-effects regression; ANOVA = analysis of variance; EMM = estimated marginal mean; SE = standard error.

We first verified the presence of mechanical allodynia and thermal hyperalgesia at the hindpaw in the neuropathic (SNI) and inflammatory (CFA) pain models using behavioural testing. Figure 2 shows painful mechanical force threshold (von Frey) and thermal withdrawal latency (Hargreaves) for both hindpaws pre- and post-treatment. In the ipsilateral (treated) hindpaw, we observed a significant increase from baseline in mechanical sensitivity for both models (*p* < .001); thermal sensitivity increased significantly for the inflammatory pain model only (*p* < .001). There were no significant changes in sensitivity on the contralateral (untreated) side.

**Figure 3.**
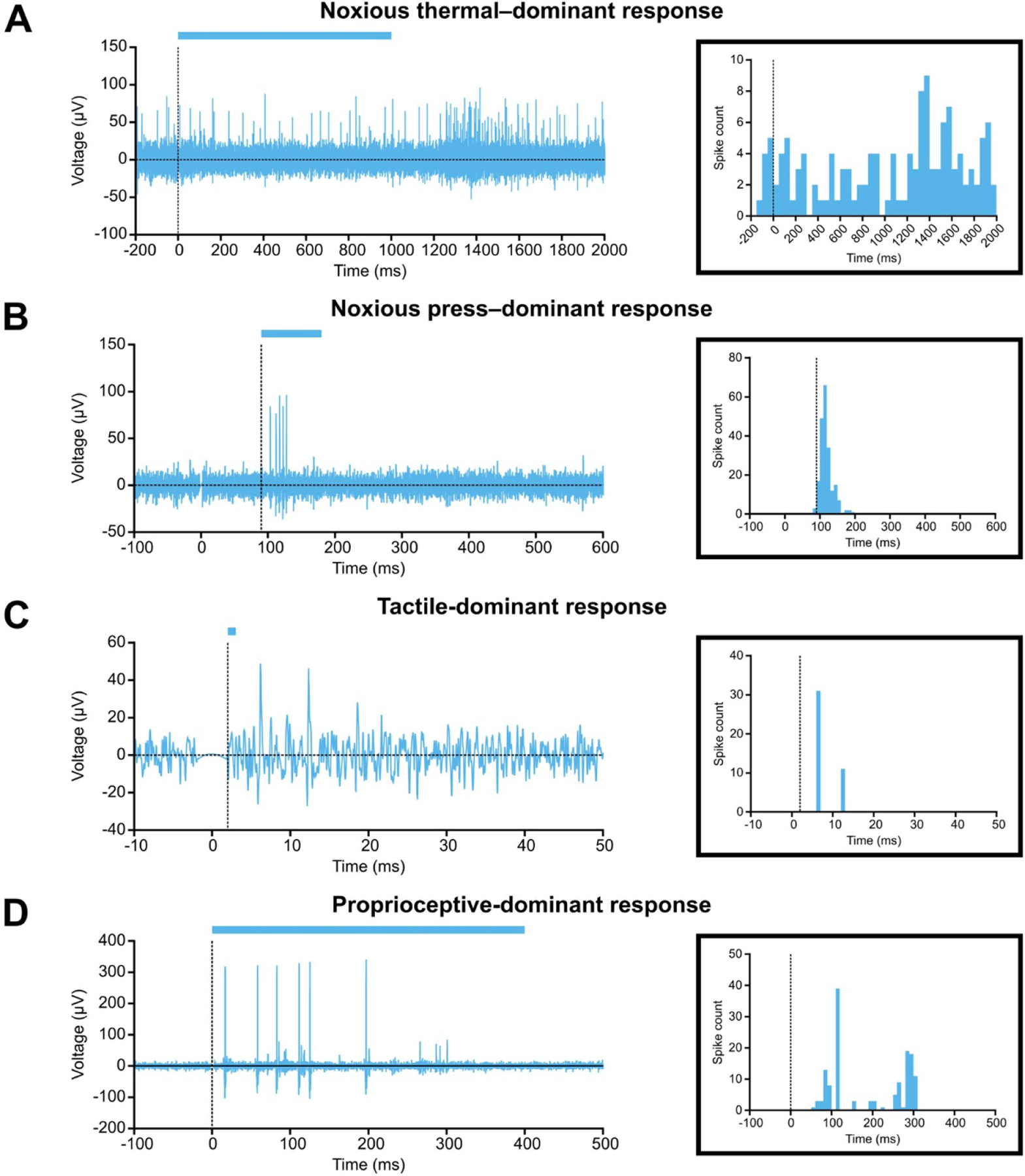
Example signal traces of each natural stimulus type. Representative dorsal horn responses to (A) noxious thermal-, (B) noxious press-, (C) tactile-, and (D) proprioceptive-dominant stimuli. Vertical dotted line indicates start of stimulus, and horizontal coloured bar represents duration of stimulus. Examples are taken from separate naïve animals. Inset: histograms of single units obtained from the corresponding trace by spike-sorting. Histogram bins are 50 ms for noxious thermal–dominant response, 10 ms for noxious press—and proprioceptive-dominant responses, and 1 ms for tactile-dominant response. Note that *Y*-axis is different for each plot to improve readability.

Having confirmed the development of pain hypersensitivity, we collected spinal recordings in response to natural stimulation of the hindpaw before, during, and after delivery of a ramped DC waveform to the sciatic nerve. We visualised recorded spike activity and selected channels for further analysis based on the presence and volume of post-stimulus spike activity. Figure 3 shows representative voltage traces in naïve animals corresponding to each of the four stimulus paradigms. The timing of each stimulus paradigm differed, as shown in blue bars above each trace, and each paradigm evoked a distinctly identifiable response pattern. Noxious thermal–dominant responses (figure 3(A)) were characterised by sustained firing and a latency of ~1 s from stimulus onset due to the extended time course of the stimulus itself. Noxious press–dominant responses (figure 3(B)) were characterised by wide but well-defined bursts of activity, with a latency of 50–100 ms from stimulus onset. Tactile-dominant responses (figure 3(C)) were typically single-or double-spike events, with highly consistent timings within 10 ms of the stimulus. Proprioceptive-dominant responses (figure 3(D)) followed a bimodal burst pattern caused by rotation and counter-rotation of the joint across 400 ms. We then spike-sorted these responses into units using principal components analysis across three channels. This yielded 563 units from naïve animals (*n* = 32 animals), 291 units from the neuropathic pain group (*n* = 8 animals), and 297 units from the inflammatory pain group (*n* = 12 animals).

### 3.1. Peripheral DC application reduces spinal cord activity evoked by pain-related stimuli in rodent models of pain

**Figure 4.**
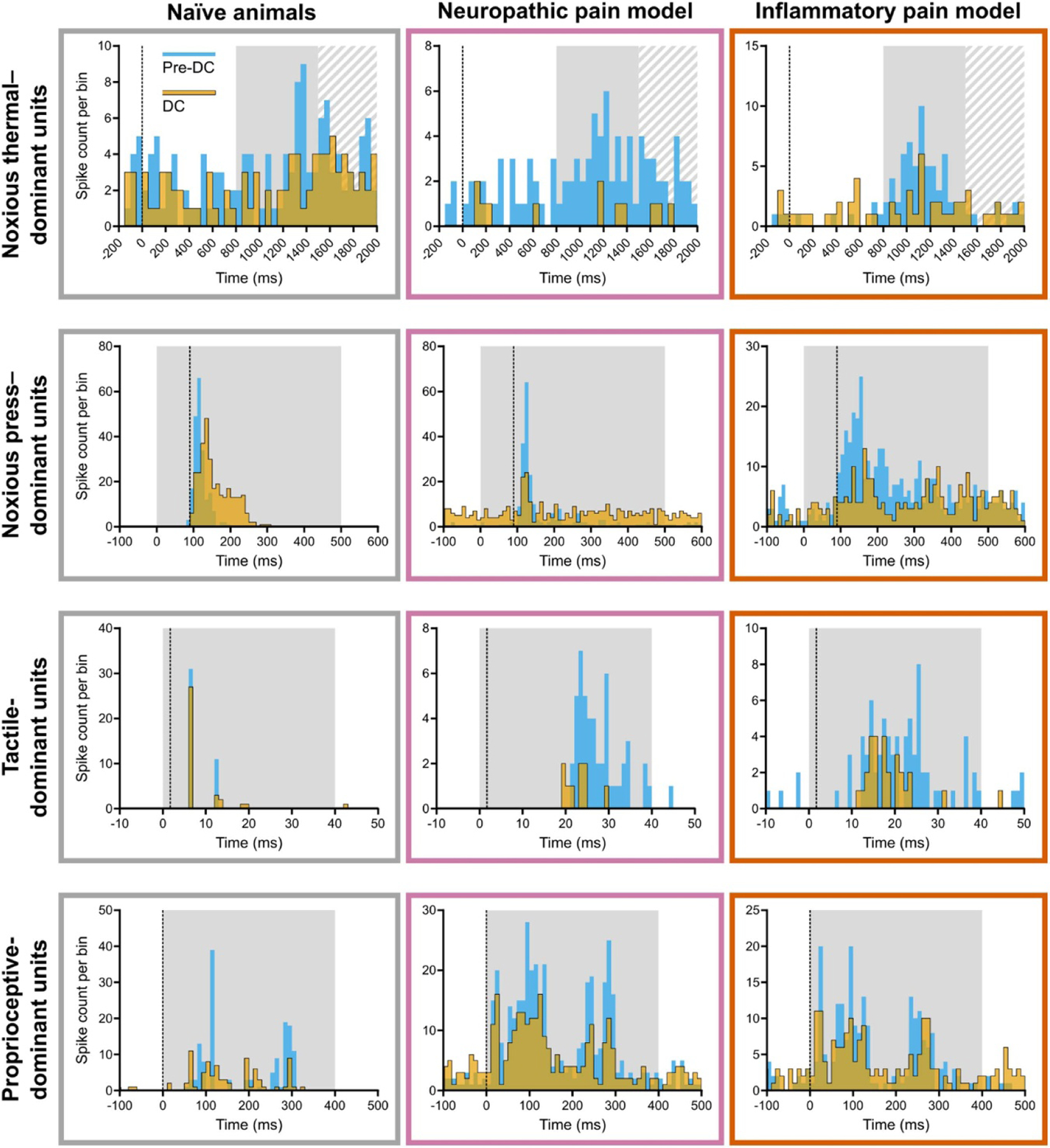
Example histograms of spike-sorted units. Histograms of spike activity during 1000 *μ*A DC (yellow) are overlaid on histograms of pre-DC baseline spike activity (blue) from the same unit. Each histogram depicts spike activity during a single recording set. Naïve recording examples are the same responses as shown in figure 3. Vertical dotted line indicates start of stimulus. Solid grey box shows post-stimulus window in which spike events were totalled for analysis and hashed grey box indicates beginning of the additional window used for analysis of sustained noxious thermal–dominant responses. Histogram bins are 50 ms for noxious thermal–dominant response, 10 ms for noxious press—and proprioceptive-dominant responses, and 1 ms for tactile-dominant response. Note that *Y*-axis is different for each plot to highlight within-panel differences rather than variance in binned peak. DC = direct current.

To determine the effects of sciatic nerve DC neuromodulation on sensory inputs to the spinal cord, we examined the activity of spike-sorted units before and after application of DC. Figure 4 shows spike count histograms of representative units during 1000 *μ*A DC (yellow) overlaid on their pre-DC baseline activity (blue). Rasters of the same units can be found in Supplementary Materials (figure S2). Each row corresponds to a natural stimulus paradigm, while each column corresponds to a treatment group. Note that to better highlight DC-mediated changes in response pattern, bin widths are consistent across each row but not between rows, and *y*-axis scale varies between each histogram. A key observation shown in this figure is that many tactile-dominant responses in pain models did not exhibit the characteristic response pattern of naïve tactile-dominant units, displaying instead a longer peak response latency (Kruskal–Wallis; *p* < .001 for neuropathic pain group, *p* = .046 for inflammatory pain group) and a non-unimodal latency distribution as indicated by Hartigans’ dip test (table 2) [52, 53].

**Figure 5.**
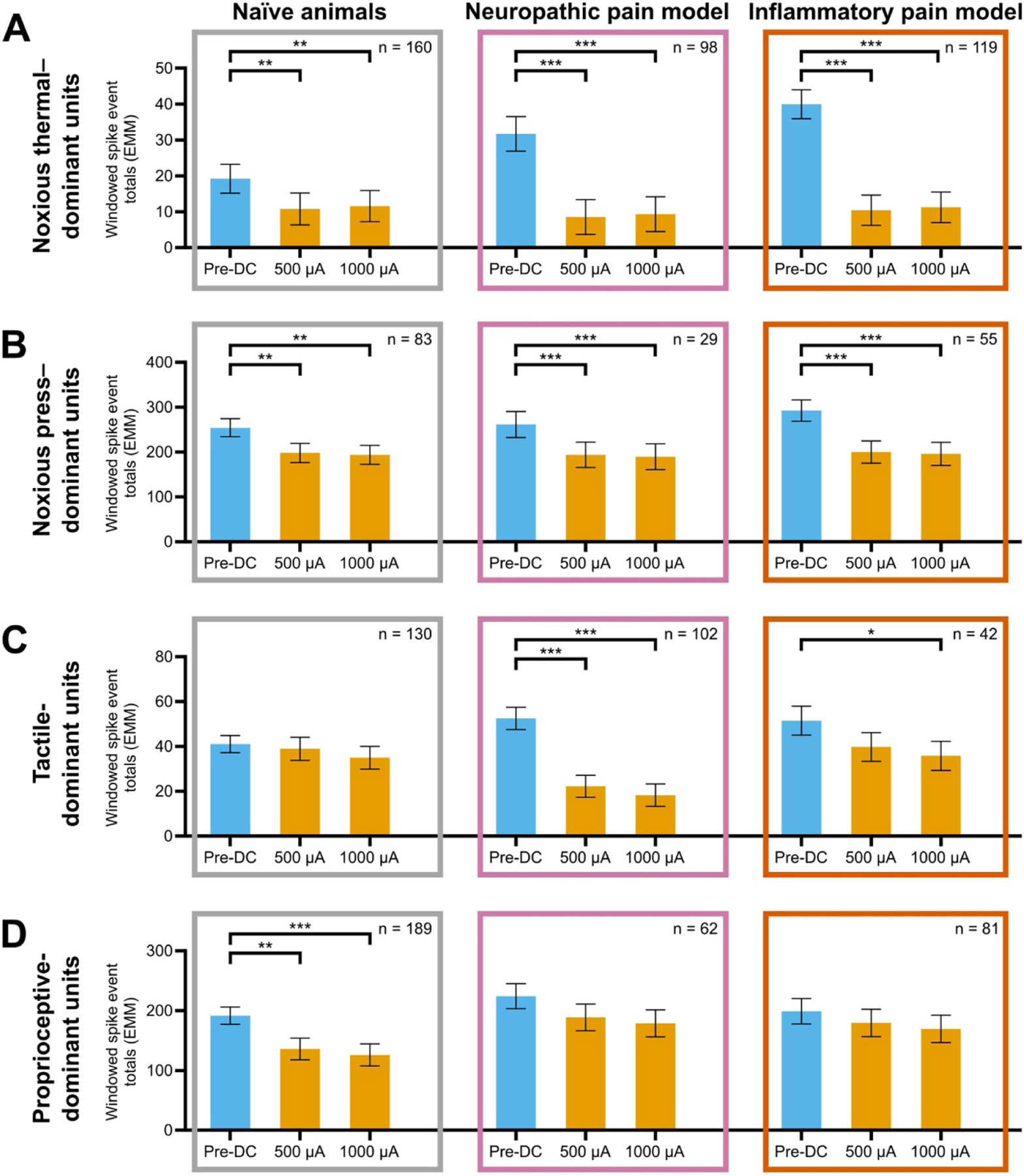
Windowed spike event totals before and during DC application. Spike totals were taken from post-stimulus windows, represented in figure 3 by solid grey boxes. EMM ± SE of these totals are shown here, as derived from LMER analyses. EMMs were compared using ANOVAs to determine significant differences. Number of units included in each comparison is provided in the respective top-right corner. Each row A-D represents a different natural stimulus modality, while each column corresponds to a treatment group. *: *p* < .05; **: *p* < .01; ***: *p* < .001. (A) Noxious thermal–dominant units. Significant differences were found between pre-DC and both DC amplitudes for all treatments (*p* < .004). Percentage reduction from baseline for these units in pain model groups were the largest of any test condition, at 70.5%–73.9%. (B) Noxious press–dominant units. Significant differences were found between pre-DC and both DC amplitudes in all treatment groups (*p* < .002). (C) Tactile-dominant units. Significant differences were found between pre-DC and 1000 *μ*A in the inflammatory pain group (*p* < .013), as well as between pre-DC and both DC amplitudes in the neuropathic pain group (*p* < .001). Reductions from baseline were relatively large in the neuropathic pain group, at 57.6%–65.2%. (D) Proprioceptive-dominant units. Significant differences were found between pre-DC and both DC amplitudes in naïve animals (*p* < .004), but not in either pain model group. DC = direct current; EMM = estimated marginal mean; SE = standard error; LMER = linear mixed-effects regression; ANOVA = analysis of variance.

In order to quantify the degree of DC-mediated reduction in activity, we totalled spike events across the response window indicated by the grey box in each histogram of figure 4. We analysed these windowed spike event totals using linear mixed-effects regressions, with significant differences determined by ANOVAs on the generated EMMs. Responses collected during application of DC at 500 *μ*A and 1000 *μ*A were compared against baseline responses prior to DC application (figure 5). No significant differences in spike totals were found between cathodic-centre and anodic-centre DC; therefore, polarity was grouped for further analysis. At 500 *μ*A and 1000 *μ*A DC, there was significant reduction in the post-stimulus activity of noxious thermal—(figure 5(A)) and noxious press–dominant (figure 5(B)) units in both pain model groups and in naïve animals (*p* < .004). At both amplitudes, there was significant reduction of tactile-dominant responses in the neuropathic pain group (figure 5(C), middle; *p* < .001) and proprioceptive-dominant responses in naïve animals (figure 5(D), left; *p* < .004). At 1000 *μ*A there was also significant reduction of tactile-dominant responses in the inflammatory pain group (figure 5(C), right; *p* = .012).

We further analysed the effect of DC-mediated reduction in activity on the sustained response to noxious thermal stimulation (1.5–4.5 s post–stimulus onset; figure S3). Figure S3(A) shows spike count histograms of representative units during 1000 *μ*A DC (yellow) overlaid on their pre-DC baseline activity (blue). Figure S3(B) shows spike event totals analysed using linear mixed-effects regressions, with significant differences determined by ANOVAs. Sustained response spike totals were significantly reduced in both pain models (*p* < .001) but not in naïve animals.

To compare the magnitude of DC-mediated reduction in activity between test conditions, we calculated the percentage reduction from baseline wherever such a change was significant. The greatest reductions from baseline were seen in noxious thermal–dominant units in pain model treatment groups (70.5%–73.9%) and tactile-dominant units in the neuropathic pain group (57.6%–65.2%). Noxious thermal—and press—dominant responses in naïve animals were reduced by 39.7%–44.0% and 22.1%–23.7% during DC application, respectively. Noxious press–dominant responses in pain model groups were reduced by 25.9%–33.0% during DC application. Tactile-dominant responses in the inflammatory pain group were reduced by 30.5% at 1000 *μ*A and proprioceptive-dominant units in naïve animals were reduced by 29.0%–34.4%. Sustained responses to noxious thermal stimulus were reduced in pain models groups by 56.4%–67.4%.

To examine the effect of DC on spontaneous activity (before application of any stimuli) in naïve animals and different pain models, we calculated both multi- (figure S4) and single-unit spike rates before and during DC application. Analysis of multi-unit activity using LMER showed an increased spike rate with DC compared to no DC application, in all treatment groups at both 500 and 1000 *μ*A (*p* < .002). Spike rates during cathodic-centre DC (EMM = 39.4 spikes/second) were higher than during anodic-centre DC (EMM = 38.9 spikes/second; *p* = .011). There were no significant differences in spontaneous spike rates between treatment groups (*p* = .281). Analysis of single-unit spike activity showed increased spontaneous spike rates at 500 *μ*A DC (EMM = 7.835 spikes/second, *p* = .013) and at 1000 *μ*A DC (EMM = 7.892 spikes/second, *p* = .001) compared to no DC application (EMM = 6.294 spikes/second) in noxious thermal units of naïve animals only, with no other significant differences.

In summary, DC neuromodulation significantly reduced spinal responses evoked by different stimuli in naïve animals compared to pain model groups. The greatest reductions in activity were observed in noxious thermal–dominant units across all treatment groups as well as tactile-dominant units in the neuropathic pain group, with smaller reductions in noxious press–dominant units in both pain model groups and proprioceptive-dominant units in naïve animals.

### 3.2. Pain-related spinal activity was reduced for longer than innocuous spinal responses following DC delivery

**Figure 6.**
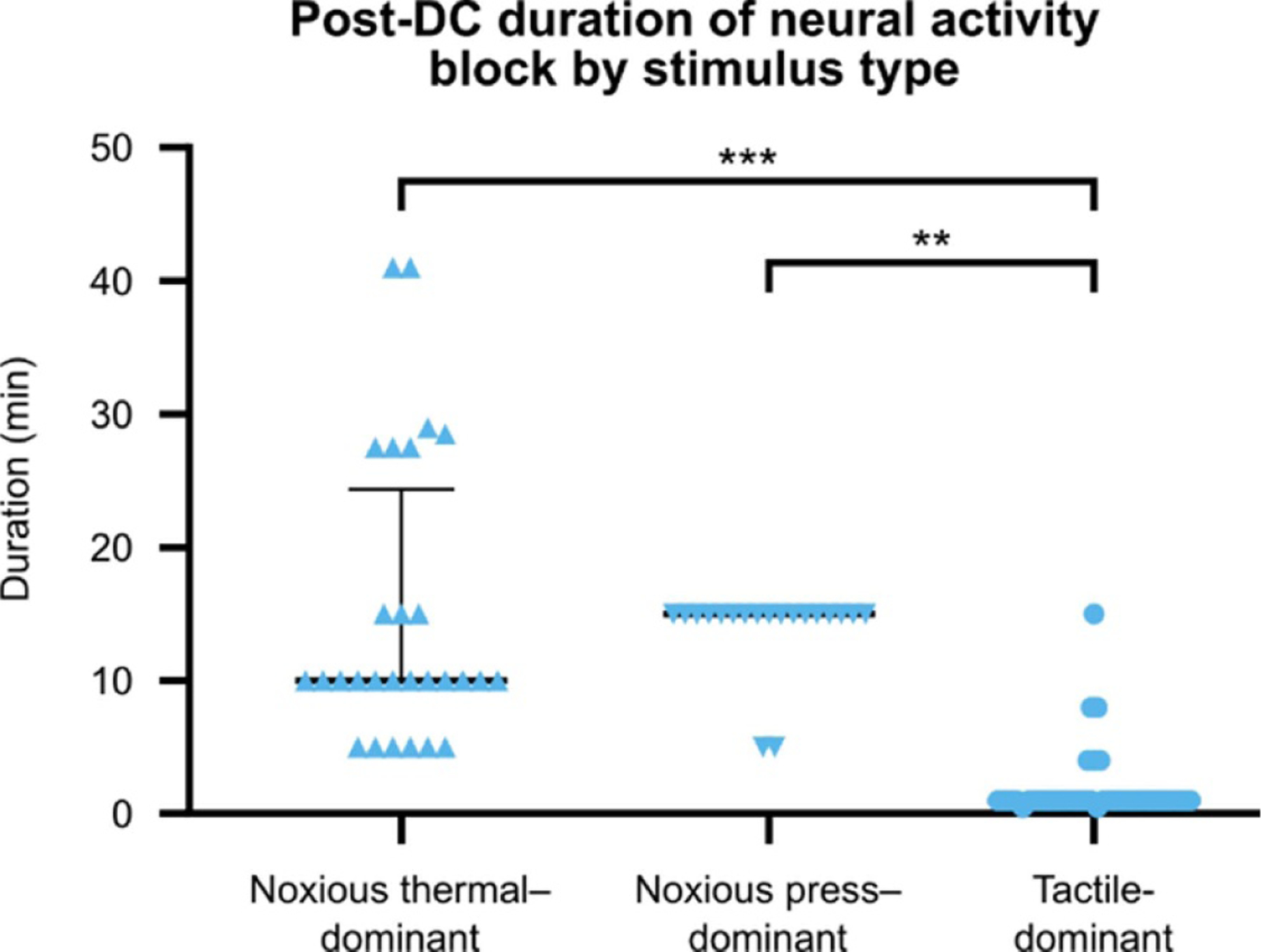
Post-DC duration of neural activity block. Time taken for evoked responses to reappear in units that ceased evoked activity during DC. This was determined by determining if evoked activity was present in post-DC recordings using a Mann–Whitney U test and Wilcoxon rank-sum post-hoc analysis with Bonferroni correction. Proprioceptive-dominant units are not shown, as all retained some level of activity during DC. Significant differences in the distribution of post-DC neural activity recovery were found between tactile-dominant units and both noxious thermal—(*p* < .001) and noxious press–dominant (*p* = .001) units. **: *p* < .01; ***: *p* < .001. DC = direct current.

Previous studies found a sustained period of activity reduction after DC neuromodulation had ended. To examine this effect in our data, we first limited the dataset to units which had no evoked activity (defined as no significant difference between pre-stimulus and post-stimulus spike counts) during application of DC. A total of 30/54 noxious thermal–, 9/30 noxious press–, and 31/115 tactile-dominant units were identified from evoked units for which post-DC recordings were made. No proprioceptive-dominant units (0/80) had no evoked activity during DC in our dataset. We defined recovery as the time to first post-DC recording which showed evoked activity in that unit. We observed recovery of activity in 28 of the 30 noxious thermal–dominant units, while all noxious press—and tactile-dominant units recovered within 15 min. We compared the duration of post-DC recovery between these responses (figure 6) and found recovery took longer in noxious thermal—(*p* < .001) and noxious pinch–dominant (*p* = .001) units than in tactile-dominant units (Mann–Whitney U). We also compared the duration of recovery to DC amplitude but found no significant difference between 500 *μ*A and 1000 *μ*A stimulus conditions (Mann–Whitney U; *p* = .350).

## 4. Discussion

The primary aim of this study was to compare the effects of peripheral DC neuromodulation on naturally evoked spinal responses in rodent models of pain. We found that evoked activity relating to both hyperalgesia and allodynia was reduced in pain models. Our results are broadly consistent with previous literature [16, 27] and provide evidence for the efficacy of peripheral DC in reducing activity associated with chronic nociceptive and chronic neuropathic pain.

We found that noxious-evoked responses (thermal and high-threshold mechanical) were consistently reduced in both pain models and in naïve animals. Noxious stimuli are associated with activity from small-diameter A*δ* and C fibres, which are not typically sensitive to traditional pulsatile neuromodulation. Previous studies reported that DC could reduce activity in these fibres at thresholds similar to those in large-diameter A*α* and A*β* fibres [16, 27]. Our results expand upon those findings in that we also demonstrate a functional reduction of nociceptive-associated activity at the spinal cord following peripheral DC neuromodulation. We achieved reduction at amplitudes roughly comparable to both Yang *et al* [16] and Jones *et al* [27]. In nociceptive responses we observed near-maximal reduction of activity at 500 *μ*A, suggesting that lower amplitudes may be maximally effective. Sustained noxious thermal–dominant responses were also reduced during DC in both pain models, but not for naïve animals. There is previous evidence to suggest that sustained responses to thermal stimulus are increased in chronic pain [51]; it is therefore possible that the effect of DC on these fibres is modified by the disease states. However, it is unclear from the present analysis whether DC is acting directly on the sustained response, or whether this reduction is the result of greater sensitivity to DC during the peristimulus response.

Our data also showed increased responses to noxious stimuli in both pain models, which may reflect hyperalgesia associated with these pain states [54]. There is some discrepancy between our electrophysiology and our behavioural verification as we did not find decreased thermal withdrawal thresholds in our neuropathic pain model. This is not unexpected, as thermal hyperalgesia does not consistently show in behavioural tests following spared tibial nerve injury, although the reason is not well understood [33, 34, 55]. Our data nonetheless suggests that some hypersensitivity is present in these models at the dorsal horn, and that these responses are reduced by peripheral DC. These results are encouraging for the use of DC neuromodulation to reduce hyperalgesic responses in both neuropathic and nociceptive chronic pain states.

In contrast, there was a less straightforward effect in innocuous-evoked responses, with reduction of tactile-dominant activity in pain models, but not in naïve animals. Chronic pain, particularly chronic neuropathic pain, contains an allodynic component wherein previously innocuous stimuli are associated with painful sensations [29]. Mechanisms of allodynia are complex, and involve pathological expression of low-threshold tactile receptors such as Piezo2 in high-threshold nociceptors as well as contribution of *C*-tactile afferents to painful sensation [56, 57]. These mechanisms alter the function of small-diameter neurons and lead to decreased pain thresholds. We found significant changes in response patterns to innocuous tactile stimuli between our naïve animals and pain models, suggesting pathological changes in the tactile-evoked neural population. This is further supported by the increased baseline response seen in our pain model groups (figure 4(C)), which displayed mechanical allodynia in behavioural tests. As such, we propose that peripheral DC may potentially reduce hypersensitive responses specifically associated with allodynia. However, further study is needed to confirm which populations of tactile-responding neurons are more strongly affected by peripheral DC.

We also observed reduction of proprioceptive-dominant responses in naïve animals, but the magnitude was smaller than for the other response types. We also did not find evidence of complete cessation of evoked activity during DC in any proprioceptive unit. We expected some effect on proprioceptive units to occur given similar findings by Jones *et al* [27], but they observed this effect in neuropathic pain models, and with complete block (i.e. cessation of evoked activity during DC; we observed only partial activity reduction in proprioceptive units), while we did not. These results reinforce that DC neuromodulation is sensitive to small changes such as stimulation site and electrode position [58, 59]. Regardless, we recommend that any future chronic stimulation study incorporates gait analysis to determine whether any potential proprioceptive effect results in functional deficit.

Our analysis of spontaneous activity revealed an increase in multi-unit activity across all treatment groups during DC delivery, with marginally higher rates for cathodic-centre DC over anodic-centre DC. This is consistent with previous reports of DC-evoked excitation [60–62]. Conversely, we did not observe increased spontaneous activity in spike-sorted units during DC across any treatment or stimulus type, except for evoked noxious thermal–dominant units in naïve animals. The multi-unit activity increase was relatively mild (*~*10%), so it is possible we did not detect this effect in our single-unit analysis as the mean firing rate was low, with many evoked units (1298 of 3821 units; 34%) not exhibiting any spontaneous firing. Nevertheless, DC-evoked excitation of any magnitude, if it continues to be observed in future studies, would need to be addressed prior to clinical trials, as increased activity could create undesirable sensations. Our lead development for ongoing studies is therefore focused on mitigating DC-evoked activity.

We did not observe any difference in pre-DC spontaneous activity in pain models compared to naïve animals. While some studies report increased activity at the dorsal horn or in peripheral nerves in animal models of pain [51, 63], others have shown no increase [64]. In the dorsal horn specifically, elevated spontaneous activity was reported from multimodal units [51, 63], which were filtered from our dataset; this may explain the discrepancy.

We did not otherwise observe significant differences between anodic-centre and cathodic-centre DC waveforms. Previous studies using monopolar DC delivery have found marked differences in responses depending on the polarity of the DC electrode [65, 66], although other studies have reported similar effects across polarities [27]. In the present study, the lack of observed differences is likely due to the tripolar design of our lead: the flanking contacts acting as returns are directly positioned adjacent to the central contact and are of opposite polarity but half amplitude. Thus, the sum effect we observe is a combination of both the central channel and the flanking returns, potentially mitigating differences between the cathodic-centre and anodic-centre stimulation waveforms. It is also possible that the stimulation amplitudes tested were too high to observe differences in polarity, which may be more distinct at low DC amplitudes [28].

Both Yang *et al* [16] and Jones *et al* [27] reported long *C* fibre recovery times following DC block, and a relationship between recovery time and block threshold has been described in the vagus nerve [67]. Jones *et al* utilised this phenomenon to develop an alternative method for selective block by delivering ultra-low frequency waveforms which allow only innocuous signalling to recover between phases. In the present study we show comparable results, with some thermal-dominant responses only returning 30 min or more after DC delivery was stopped. This provides further evidence to support their approach. However, it reveals a potential confound in our study design. Although we show long time courses for recovery, sequential recordings in multiple locations are made over the course of an experiment. As the DC waveform was delivered to the entire sciatic nerve, it is possible that its effects persisted when starting a new set of recordings. We controlled for this by leaving long recovery periods and always comparing against pre-DC baseline activity. We cannot completely rule out the possibility of longer duration effects of DC neuromodulation, but their analysis is beyond the scope of this study.

It is also important to note that our results are unlikely to be explained by nerve block mechanisms alone. The majority of units analysed, including those with significant reductions in activity, continued to produce evoked responses during both amplitudes of DC tested. As we did not standardise DC amplitude to block threshold and instead gave fixed amplitudes, it is our expectation that some units in the dataset were excited while the activity of others was reduced. Furthermore, subthreshold DC modulates both neural sensitivity and conduction velocity [58, 68–70] and our data does not preclude desynchronisation of peripheral inputs as an explanation for our findings. Our analysis of the overall response under these conditions more accurately reflects the realities of implantable devices, which cannot be calibrated to per-neuron block thresholds for practical reasons. However, a limitation of our methodology is that it does not provide much insight into the underlying mechanisms of action. Additionally, our study does not examine the dose-response curve of DC-mediated spinal activity reduction. However, as that relationship is likely to differ in humans compared to rats, it would be more informative to investigate that relationship in future animal behavioural studies followed by human clinical trials.

## 5. Conclusion

By demonstrating reduction of functional, pain-related activity in both neuropathic and inflammatory nociceptive pain models, we highlight an advantage of peripheral DC neuromodulation over traditional pulsatile stimulation. There is limited evidence for the efficacy of existing devices in the management of chronic nociceptive pain conditions [13, 14], such as post-surgical or arthritic pain. DC neuromodulation could be particularly valuable for the treatment of these conditions and to help minimise risks associated with chronic opioid use [5, 71]. Our next steps in developing this technology include designing a chronically implantable device with which we will test long-term implantable lead safety. In parallel, we will perform awake behaving animal experiments to further validate functional reduction of nociception and pain. Another avenue for future investigation is to explore the mechanisms driving these observations. Previous findings have shown that, in addition to conduction block, DC neuromodulation can promote input desynchronisation and post-synaptic inhibition [58, 68]. It is therefore necessary for future studies to determine whether DC waveforms directly block action potential propagation in peripheral fibres or mediate inhibition via more complex central mechanisms. Overall, our results support the continued translation of peripheral DC neuromodulation as a potential treatment for chronic pain.

## Supplementary Material

Supplementary Material

## Figures and Tables

**Table 1.** Window timings for analysis of spike-event totals. Spikes within these pre-and post-stimulus windows were counted for use in statistical analysis.

	Pre-stimulus window (ms after trigger)	Post-stimulus window (ms after trigger)
Noxious thermal-dominant units	−700–0	800–1500
Noxious thermal-dominant units (sustained response)	−1000–0	1500–4500
Noxious press-dominant units	−500–0	0–500
Tactile-dominant units	−40–0	0–40
Proprioceptive-dominant units	−400–0	0–400

**Table 2.** Distribution of response latencies for tactile-dominant units by treatment group. Distribution is determined using Hartigans’ dip test for unimodality [52]. Interpretations of Hartigans’ dip test *p*-values are from Freeman and Dale [53].

	Mean latency (ms)	Hartigans’ dip test
Naïve animals	8.31	*p* = .156 (unimodal)
Neuropathic pain model	10.85	*p* = .034 (polymodal)
Inflammatory pain model	10.41	*p* = .074 (weakly polymodal)

## Data Availability

The data that support the findings of this study are openly available at the following URL/DOI: https://github.com/Aplinlab/Su-et-al.-2024-Data.
