# Supplementary material for: Peripheral direct current reduces naturally evoked nociceptive activity at the spinal cord in rodent models of pain
Source: J Neural Eng. Author manuscript; Available in PMC 2025 Aug 17. (PMC12358189; doi:10.1088/1741-2552/ad3b6c)
Supplement: Supplementary Material [file NIHMS2099030-supplement-Supplementary_Material.pdf]

Peripheral direct current reduces naturally evoked nociceptive activity at the spinal cord in rodent models of pain

Tom F. Su, Jack D. Hamilton, Yiru Guo, Jason R. Potas, Mohit N. Shivdasani, Gila Moalem-Taylor, Gene Y. Fridman, Felix P. Aplin

Supplementary materials

This file includes supplementary figures S1-4.

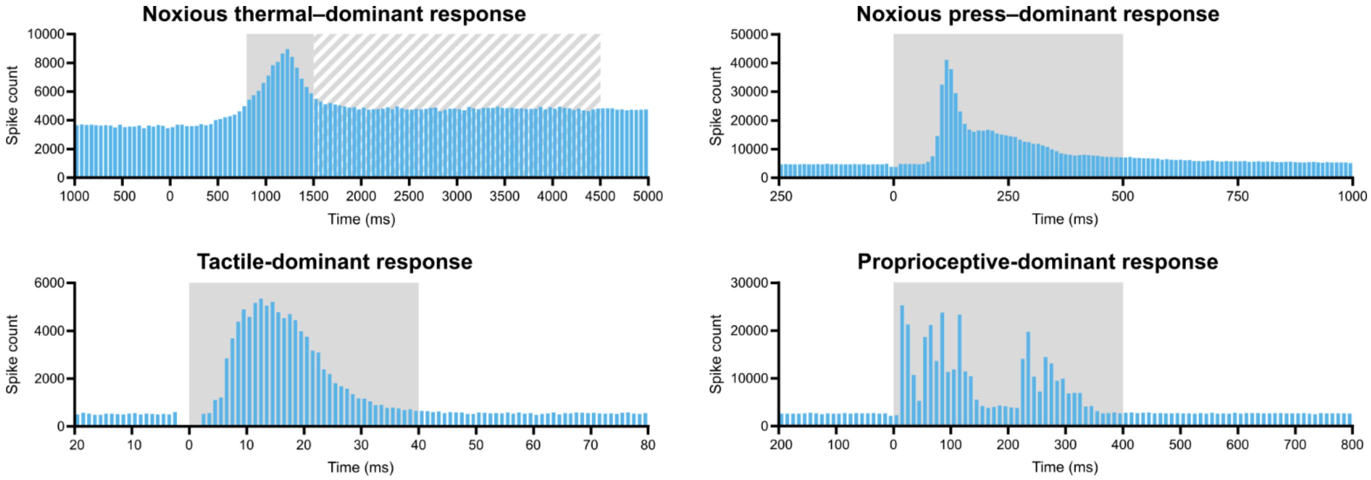

Figure S1. Aggregate firing rates over time. Histograms show the summed spike events over time across all channels selected for data analysis. Windows for analysis of spike-sorted units (solid grey boxes) were determined using a threshold of 1.5 times pre-stimulus baseline rate. Window boundaries were rounded outwards to 1 significant figure. An additional 3 s window was used during analysis of noxious thermal–dominant units to examine the sustained response (hashed grey box). Note that the x-axis range is different for each of the above histograms, reflecting the difference in response pattern to each stimulus type.

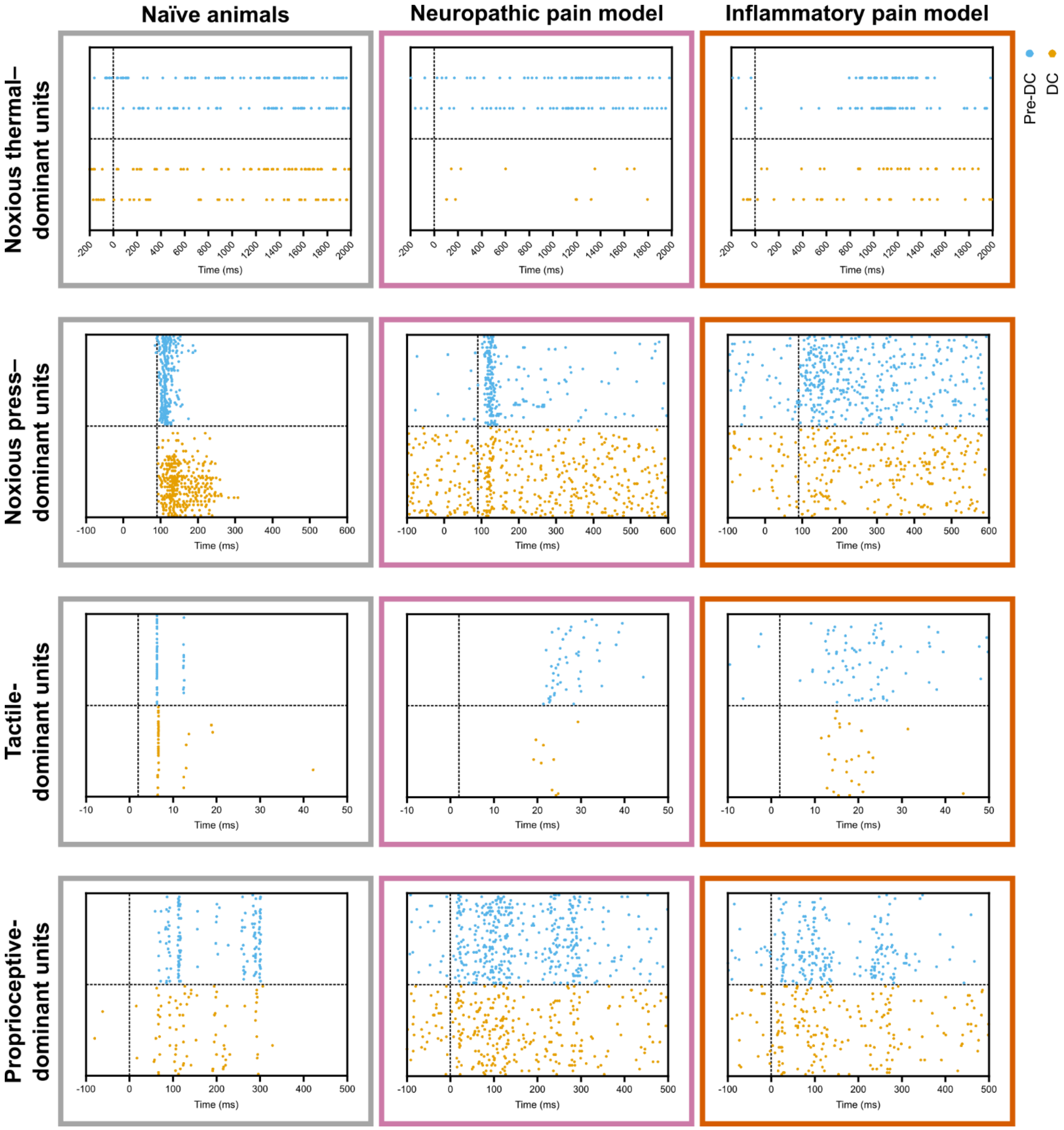

Figure S2. Example rasters of spike-sorted units. Rasters of pre-DC baseline spike activity (blue) and spike activity during 1000 μA DC (yellow). This figure displays the same data as figure 4, with each raster depicting spike activity during a single pre-DC recording set and a single during-DC recording set. Baseline–DC pairs are taken from the same unit. Each unit was recorded from a different animal. Vertical dotted line indicates start of stimulus. DC = direct current.

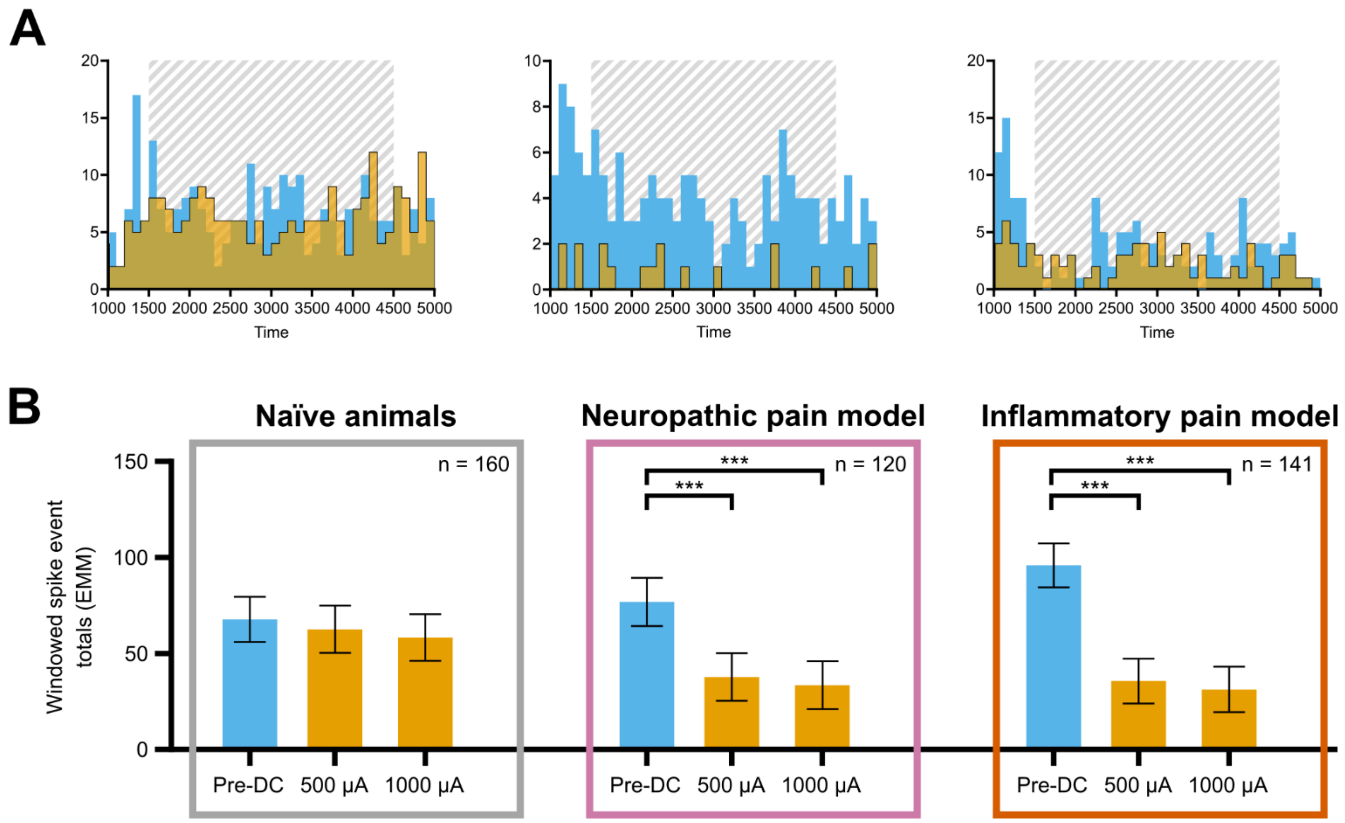

Figure S3. Effects of DC on the sustained response to noxious thermal–dominant stimulus. (A) Example histograms of spike-sorted units. Histograms of recordings during 1000 µA DC (yellow) are overlaid on histograms of pre-DC baseline recordings (blue) of the same unit. Note that y-axis is different for each plot to highlight within-panel differences rather than variance in binned peak. (B) Windowed spike event totals before and during DC application. Spike event totals were taken from post-stimulus windows shown in (A). EMM ± SE of these totals are shown here, as derived from LMER analyses. EMMs were compared using ANOVAs to determine significant differences. Number of units included in each comparison is provided in the respective top-right corner. The sustained response to noxious thermal–dominant stimulus was significantly reduced during application of direct current at 500 µA and 1000 µA ($p < 0.001$) in both pain models, but not in naïve animals. *** : $p < .001$. DC = direct current; EMM = estimated marginal mean; SE = standard error; LMER = linear mixed-effects regression; ANOVA = analysis of variance.

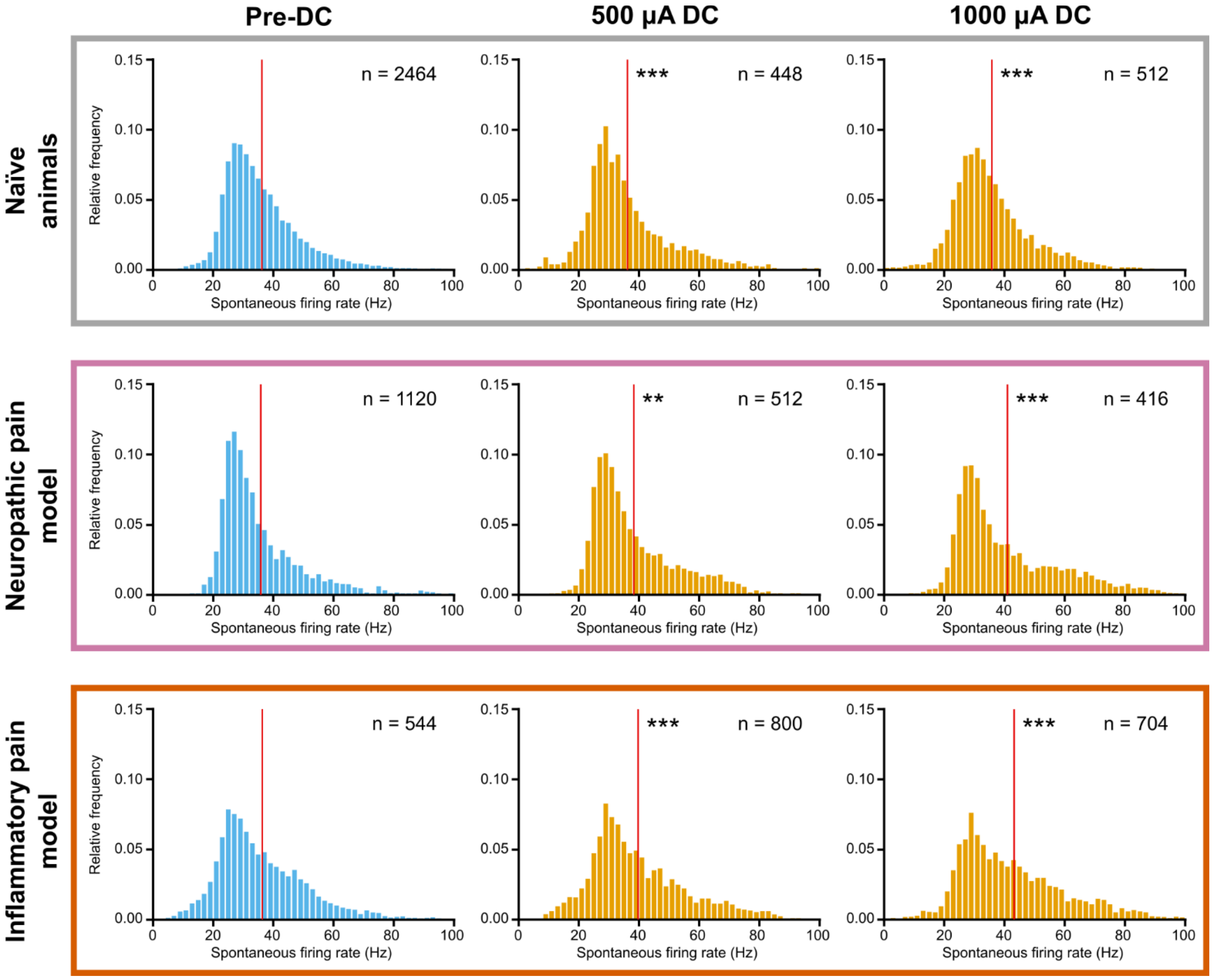

Figure S4. Frequency distributions of multi-unit spontaneous firing rate before and during DC. Histograms show the relative frequency of per-channel multi-unit firing rates (in spikes/second) across treatment groups and DC amplitudes. The EMMs of firing rates are shown as red lines and number of channels included in each histogram is provided in the respective top-right corner. Data were analysed using a LMER with fixed effects including treatment group and DC amplitude, and nested random effects including animal and recording identifiers. Significance was determined by comparing EMMs by ANOVA. The mean firing rate during 500 µA or 1000 µA DC was found to be significantly higher than pre-DC ($p < .002$). Note that the area under the curve has been normalised to 1. Asterisks represent significant differences when comparing mean firing rates against pre–direct current mean firing rates. ** : $p < .01$; *** : $p < .001$. DC = direct current; EMM = estimated marginal mean; LMER = linear mixed-effects regression; ANOVA = analysis of variance.
